# Adverse Effects of Stimulant Interventions for Attention Deficit Hyperactivity Disorder (ADHD): A Comprehensive Systematic Review

**DOI:** 10.7759/cureus.45995

**Published:** 2023-09-26

**Authors:** Ankita Nanda, Lakshmi Sai Niharika Janga, Hembashima G Sambe, Mohamed Yasir, Ruzhual K Man, Amaresh Gogikar, Lubna Mohammed

**Affiliations:** 1 Research, California Institute of Behavioral Neurosciences & Psychology, Fairfield, USA; 2 Internal Medicine, California Institute of Behavioral Neurosciences & Psychology, Fairfield, USA

**Keywords:** attention deficit hyperactivity disorder (adhd), lisdexamfetamine dimesylate, amphetamines, stimulants, methylphenidate

## Abstract

Attention deficit hyperactivity disorder (ADHD) is a fairly common psychiatric disorder among children. It has substantial consequences in terms of quality of life for those experiencing it and their families. In managing ADHD symptoms medication plays an essential role, including stimulants such as methylphenidate being a key component. Nevertheless, concerns have been raised about possible adverse reactions connected to these drugs. Thus, in this systematic review, an extensive analysis was conducted aiming at understanding any negative repercussions specifically from prolonged exposure to these medications among patients diagnosed with ADHD.

The methodology entailed adhering to the Preferred Reporting Items for Systematic Reviews and Meta-Analyses (PRISMA) 2020 guidelines. While capturing relevant data through a meticulous search in various databases, filtered according to preset inclusion and exclusion criteria, 13 studies were considered for analysis. Conclusions indicate that the administration of stimulant medications can potentially translate into a small rise in blood pressure along with increased heart rate particularly when amphetamines are taken. However, no reports of notable serious cardiovascular events have emerged. In the domain of neuropsychiatry, it appears that long-term usage of methylphenidate generally bears no serious consequences, even though a hike in risk levels related to the occurrence of psychotic episodes was detected among those treated with amphetamines. Several gastrointestinal side effects including decreased appetite and stomach pain were reported, however, findings regarding ocular abnormalities or growth-related effects stood inconclusive.

Therefore, based on this data the consensus is that stimulant medications do generate manageable and mild negative outcomes within the ADHD population. It is vital however to highlight the need for careful observation and further scientific inquiry to achieve a better grasp on both immediate as well as long-term implications involved.

## Introduction and background

Attention deficit hyperactivity disorder (ADHD) is a prevalent childhood psychiatric disorder that affects approximately 3-5% of school-age children globally [1]. This condition is characterized by two primary symptom dimensions: inattention and motor restlessness/impulsivity. These dimensions have a widespread impact and significantly limit a child’s functioning [2]. Studies have indicated that a significant percentage up to 70% of individuals diagnosed with ADHD during childhood continue to experience symptoms even through their adult years [3]. Research also suggests that boys are three times more likely to be diagnosed with ADHD compared to girls [4]. Additionally, among the different types of ADHD, the hyperactive impulsive type is commonly observed in boys while the inattentive type is more prevalent among girls [5].

ADHD has negative effects on various aspects of a child’s life, including social interactions, academic performance, and family dynamics. Thus, it is crucial to identify and address ADHD early on to enhance overall outcomes for children and their families [6]. A considerable proportion (around 18-50%) of individuals who start treatment for ADHD continue taking medication for an extended period, usually two to three years [7].

The management of ADHD typically involves a comprehensive approach that combines both pharmacological and non-pharmacological interventions [8]. Stimulants are considered the initial treatment of choice and have been used for over five decades to effectively address ADHD symptoms by improving attention span, reducing distractibility, enhancing memory function, minimizing impulsivity, and mitigating hyperactivity [9-11]. In addition, guanfacine extended-release (XR) and clonidine XR were the only drugs recognized by the US Food and Drug Administration (FDA) for supplementary therapy in stimulant-treated children and adolescents with ADHD [12].

All the stimulants belong to a class called sympathomimetic agents and have similar chemical compositions and physiological actions. They work by stimulating the central nervous system, leading to increased levels of noradrenaline and dopamine (DA) in the prefrontal cortex. Additionally, they activate adrenergic receptors in the heart and blood vessels which results in slight elevations in resting heart rate (RHR) and blood pressure (BP) [13]. That’s the reason, among all the available medications for ADHD management, CNS stimulants pose the greatest risk when it comes to cardiovascular (CV) complications [14]. Thus, it is crucial to determine whether or not observed CV impacts of ADHD medication in a larger population have significant implications for certain individuals, potentially raising their risk for severe CV events like stroke or sudden death [1].

Aside from the concerns raised by the media, various stakeholders including interest groups, scientists, and health professionals have also expressed apprehension about a wide range of potential negative effects linked to the long-term use of stimulant treatments [15]. Children and adolescents taking these medications may experience different side effects such as reduced appetite, increased aggression, and physical symptoms like headaches and gastrointestinal issues [6]. Additionally, due to the dopaminergic activity of stimulant medications, long-term exposure has the potential to increase the risk of psychosis, as supported by existing literature [16]. Investigating the relationship between long-term stimulant treatment and negative neuropsychiatric effects is undoubtedly significant from a clinical standpoint but it poses a complex challenge in terms of finding definitive answers. One crucial factor that should be taken into account is the severity of ADHD as it could serve as a confounding variable since it may be associated with both the requirement for long-term medication therapy and substantial underlying neuropsychiatric comorbidity [16].

The objective of our present study is to thoroughly analyze existing evidence encompassing adverse effects, including behavioral effects, associated with stimulant treatment for ADHD. We aim to assess potential adverse outcomes based on the included studies.

## Review

Methodology

In line with the guidelines presented in the Preferred Reporting Items for Systematic Reviews and Meta-Analyses (PRISMA) 2020, we conducted this systematic review [17].

Eligibility Criteria

To choose the final studies for the review, specific criteria were employed. This included the requirement that only articles offering unrestricted access to their full text were considered for evaluation. Eligibility was restricted to studies published exclusively in the English language between the years 2013 and 2023. The review was focused on studies which included both males and females, aged between 0 and 25 years. It encompasses both review articles and research articles. However, studies lacking full-text availability, those published in languages other than English, those published before 2013, studies involving age groups older than 25 years, as well as research reporting on animal experiments, were deemed ineligible for inclusion in the review.

Search Strategy and Databases

In our search for potential publications on the adverse effects of stimulants in ADHD, we utilized several databases, namely PubMed/Medline, PubMed Central (PMC), Google Scholar, Science Direct, and Cochrane. To ensure a comprehensive search, we employed a combination of Medical Subject Heading (MeSH) phrases and keywords using the Boolean technique. The specific keywords used included attention deficit hyperactivity disorder (ADHD), Methylphenidate, Stimulants, Amphetamines, and Lisdexamfetamine Dimesylate. To streamline the process, we utilized the EndNote reference manager to compile the references and eliminate any duplicates. Subsequently, studies were screened based on their titles and abstracts to exclude irrelevant ones. Finally, the remaining full-text papers were thoroughly reviewed for quality evaluation. The search strategy’s detailed information can be found in Table 1 [18].

**Table 1:** Information on the Search Strategy Used, Including the Specific Filters PMC: PubMed Central; ADHD: attention deficit hyperactivity disorder

Search Strategy	Database Used	Number of Research Papers
( "Attention Deficit Disorder with Hyperactivity/complications"[Majr] OR "Attention Deficit Disorder with Hyperactivity/diagnosis"[Majr] OR "Attention Deficit Disorder with Hyperactivity/diet therapy"[Majr] OR "Attention Deficit Disorder with Hyperactivity/drug therapy"[Majr] OR "Attention Deficit Disorder with Hyperactivity/etiology"[Majr] OR "Attention Deficit Disorder with Hyperactivity/genetics"[Majr] OR "Attention Deficit Disorder with Hyperactivity/metabolism"[Majr] OR "Attention Deficit Disorder with Hyperactivity/pathology"[Majr] OR "Attention Deficit Disorder with Hyperactivity/physiopathology"[Majr] OR "Attention Deficit Disorder with Hyperactivity/prevention and control"[Majr] OR "Attention Deficit Disorder with Hyperactivity/therapy"[Majr] ) AND ( "Methylphenidate/administration and dosage"[Majr] OR "Methylphenidate/adverse effects"[Majr] OR "Methylphenidate/agonists"[Majr] OR "Methylphenidate/antagonists and inhibitors"[Majr] OR "Methylphenidate/blood"[Majr] OR "Methylphenidate/metabolism"[Majr] OR "Methylphenidate/pharmacokinetics"[Majr] OR "Methylphenidate/pharmacology"[Majr] OR "Methylphenidate/therapeutic use"[Majr] OR "Methylphenidate/toxicity"[Majr] ) OR ( "Amphetamines/administration and dosage"[Majr] OR "Amphetamines/adverse effects"[Majr] OR "Amphetamines/agonists"[Majr] OR "Amphetamines/antagonists and inhibitors"[Majr] OR "Amphetamines/blood"[Majr] OR "Amphetamines/metabolism"[Majr] OR "Amphetamines/pharmacokinetics"[Majr] OR "Amphetamines/pharmacology"[Majr] OR "Amphetamines/therapeutic use"[Majr] OR "Amphetamines/toxicity"[Majr] )	PubMed	371
( "Attention Deficit Disorder with Hyperactivity/complications"[Majr] OR "Attention Deficit Disorder with Hyperactivity/diagnosis"[Majr] OR "Attention Deficit Disorder with Hyperactivity/diet therapy"[Majr] OR "Attention Deficit Disorder with Hyperactivity/drug therapy"[Majr] OR "Attention Deficit Disorder with Hyperactivity/etiology"[Majr] OR "Attention Deficit Disorder with Hyperactivity/genetics"[Majr] OR "Attention Deficit Disorder with Hyperactivity/metabolism"[Majr] OR "Attention Deficit Disorder with Hyperactivity/pathology"[Majr] OR "Attention Deficit Disorder with Hyperactivity/physiopathology"[Majr] OR "Attention Deficit Disorder with Hyperactivity/prevention and control"[Majr] OR "Attention Deficit Disorder with Hyperactivity/therapy"[Majr] ) AND ( "Methylphenidate/administration and dosage"[Majr] OR "Methylphenidate/adverse effects"[Majr] OR "Methylphenidate/agonists"[Majr] OR "Methylphenidate/antagonists and inhibitors"[Majr] OR "Methylphenidate/blood"[Majr] OR "Methylphenidate/metabolism"[Majr] OR "Methylphenidate/pharmacokinetics"[Majr] OR "Methylphenidate/pharmacology"[Majr] OR "Methylphenidate/therapeutic use"[Majr] OR "Methylphenidate/toxicity"[Majr] )	PMC	171
allintitle: ADHD AND Methylphenidate OR Amphetamines	Google Scholar	330
ADHD AND Amphetamines OR Methylphenidate OR Lisdexamfetamine dimesylate	Science Direct	293
ADHD AND Methylphenidate OR Amphetamines	Cochrane	30

Results

Study Selection and Quality Evaluation

Before April 20, 2023, a total of 1195 references were retrieved from five databases: PubMed, PubMed Central, Science Direct, Google Scholar, and Cochrane. After removing 69 duplicate references, there were 1126 unique records left. By conducting a thorough screening of titles and abstracts, 1080 irrelevant records were excluded, resulting in 46 potentially relevant references. After a full-text screening of these 46 references, 28 more records were excluded, leaving a final selection of 18 references.

**Figure 1:**
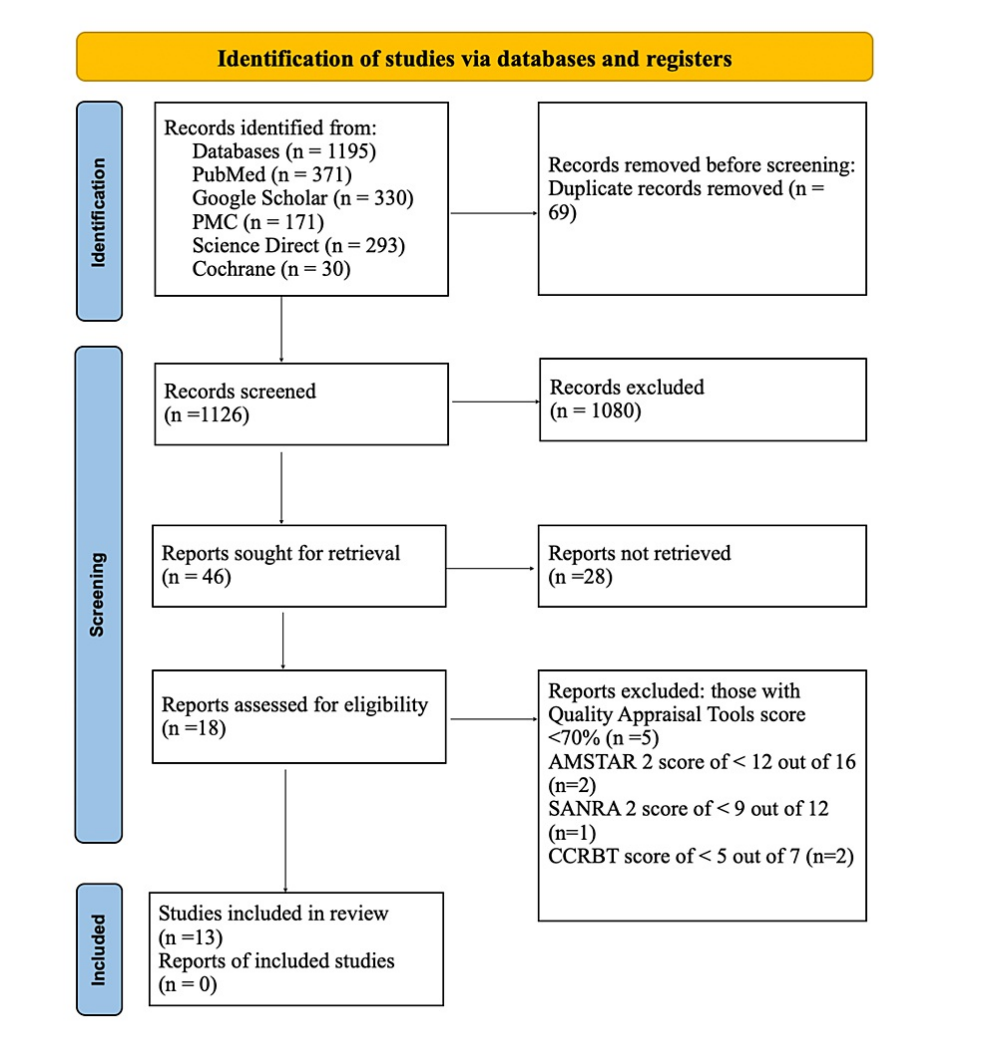
Prisma Flowchart AMSTAR 2: Assessment of Multiple Systematic Reviews 2; SANRA 2: Scale for the Assessment of Narrative Review Articles 2; CCRBT: Cochrane Collaboration Risk of Bias Tool The flowchart was created by authors’ team.

Subsequently, a quality assessment was performed using specialized instruments for each study type, leading to the inclusion of 13 high-quality studies with a score of over 70%. The final research study consisted of six systematic reviews, five cohort studies, one randomized control trial (RCT), and one traditional review [18]. Figure 1 displays a flow diagram illustrating the search and selection process.

Study Characteristics

A thorough summary and in-depth examination of the process of quality assessment, along with the specific tools used to evaluate the selected articles for the review is presented in Table 2.

**Table 2:** Detailed Analysis of the Quality Assessment Procedure and the Methods and Tools Used to Analyze the Reviewed Articles CCRBT: Cochrane Collaboration Risk of Bias Tool; NCOT: New Castle Ottawa Tool; AMSTAR 2: Assessment of Multiple Systematic Reviews 2; SANRA 2: Scale for the Assessment of Narrative Review Articles 2; RCTs: randomized controlled trials

Quality Assessment Tool	Type of Study	Total Score	Accepted Score (>70%)	Number of Accepted Studies (#)
NCOT	Observational Studies	9	7	5; Kamal et al. (2018) [6], Omidi et al. (2021) [14], Moran et al. (2019) [19], Bingöl-Kızıltunç et al. (2022) [20], Poulton et al. (2016) [21]
AMSTAR 2	Systematic Review, Meta-Analysis	16	12	6; Hennissen et al. (2017) [1], Carucci et al. (2021) [2], Krinzinger et al. (2019) [16], Torres-Acosta et al. (2020) [22], Holmskov et al. (2017) [23], Storebø et al. (2018) [24]
SANRA 2	Narrative Review	12	9	1; Reiersen (2018) [25]
CCRBT	RCTs	7	5	1; Sayer et al. (2016) [26]

A summary of the main characteristics of the observational studies included in the analysis is presented in Table 3.

**Table 3:** Major Features of the Observational Studies Included in the Study ADHD: attention deficit hyperactivity disorder; DSM: Diagnostic and Statistical Manual of Mental Disorders; MPH: methylphenidate

First Author, Year	Study Type	Inclusion Criteria	Sample Size	Outcome
Kamal et al. (2018) [6]	Prospective cohort study	Patients who fulfilled the diagnostic criteria for ADHD according to the DSM-V and had inattention, behavioral problems, hyperactivity, impulsivity, and/ or aggressive behavior along with inattention affected academic difficulties due to ADHD.	101	The data revealed that more than half of the patients experienced at least one side effect; however, most patients reported that intervention with medication had a positive impact, with marked improvement in academics and behavior.
Omidi et al. (2021) [14]	Prospective cohort study	Children aged between 6 and 11 years, with the diagnosis of ADHD using DSM-V criteria referred to the psychiatric clinic of Ziaeian Hospital in Tehran, Iran.	100	Apart from a non-pathological increase in all indices after 3-month follow-up, short-term (3-month) treatment of ADHD children with CNS stimulants doesn’t show any statistically significant relationship with altered blood pressure indices.
Moran et al. (2019) [19]	Prospective cohort study	Patients between 13 and 25 years of age who had one or more than one out-patient encounters with the diagnosis of ADHD and started treatment with amphetamine or methylphenidate between January 2004 to September 2015.	221,846	The incidence of new-onset psychosis in adolescents and young adults with ADHD, who are on prescription stimulants, is approximately one in 660 patients. In addition, it was found that amphetamine was more associated with psychosis than methylphenidate.
Bingöl-Kiziltunc (2022) [20]	Prospective cohort study	Children newly diagnosed with ADHD according to the DSM-V criteria were included in the study.	22	The data indicates that there may be a relationship between the structural and ocular parameters, especially accommodation capacity, and methylphenidate treatment.
Poulton et al. (2016) [21]	Prospective cohort study	Stimulant naive children <12 years of age with the diagnosis of ADHD according to the DSM-IV criteria were included.	73	Children treated for ADHD had growth delay which was medication dose related and could likely increase the prevalence of short stature in children with ADHD.

A summary of the main attributes of the systematic reviews that were incorporated into the analysis is presented in Table 4.

**Table 4:** An Overview of the Key Features of the Systematic Reviews Used in the Analysis ADHD: attention deficit hyperactivity disorder; MPH: methylphenidate; AMP: amphetamine; ATX: atomoxetine; HR: heart rate; SBP: systolic blood pressure; DBP: diastolic blood pressure; DSM: Diagnostic and Statistical Manual of Mental Disorders; CVD: cardiovascular diseases; RHR: resting heart rate; NR: narrative review; IQ: intelligence quotient

First Author, Year	Study Type	Inclusion Criteria	Outcomes
Hennissen et al. (2017) [1]	Systematic review	Studies in which patients diagnosed with ADHD, 0-18 years of age treated with MPH, AMP, or ATX and got their cardiovascular parameters like HR, SBP, and DBP measured at baseline were included.	Review indicated that AMP and ATX are associated with statistically significant increase in pre-, post-DBP, SBP, and HR whereas MPH had shown no significant effects.
Carucci et al. (2021) [2]	Systematic review	The included studies were written in English language and provided data on human participants diagnosed with ADHD according to DSM-III, DSM-III-R, or DSM-IV criteria, who received treatment with methylphenidate for a minimum duration of 6 months.	Methylphenidate causes mild reduction in height and weight when administered as a long-term therapy but these changes have a minimal clinical impact.
Krinzinger et al. (2019) [16]	Systematic review	Original full-text articles which provided evidence regarding the neurological, psychiatric, and behavioral effects of MPH treatment in participants diagnosed with ADHD were included.	The review suggests that long-term MPH is not associated with adverse neuropsychiatric outcomes. Moreover, it might reduce depression and suicide in ADHD patients. However, evidence suggests caution in specific groups including preschool children, those with tics, and adolescents at risk for substance misuse.
Torres-Acosta et al. (2020) [22]	Systematic review	The review encompasses studies discussing the potential cardiovascular risks of ADHD medications, as well as articles examining non-pharmacological approaches for managing ADHD.	According to the review, the use of stimulant medications raises the chances of CVDs and is linked to an increase in RHR and BP. Omega-3 fatty acids and regular physical exercise are suggested as alternative treatments.
Holmskov et al. (2017) [23]	Systematic review	The review included randomized clinical trials describing children and adolescents diagnosed with ADHD according to DSM criteria.	The administration of methylphenidate to children and adolescents diagnosed with ADHD is associated with an elevated likelihood of experiencing reduced appetite, weight loss, and abdominal discomfort.
Storebø et al. (2018) [24]	Systematic review	Studies that spoke about methylphenidate as an intervention for ADHD were included. The participants had to be less than 20 years, diagnosed with ADHD according to DSM III-V criteria, and at least 75% of them had to have normal intellect (IQ > 70).	It was concluded that methylphenidate was associated with numerous serious and nonserious adverse effects which might cause the children and adolescents taking it to withdraw the medication.
Reiersen (2018) [25]	Traditional review	NR	Methylphenidate is associated with treatment-emergent psychotic symptoms in 1.1-2.5% of the people who take it as therapy for ADHD.

Essential characteristics of the randomized clinical trials that were considered in the analysis are outlined in Table 5.

**Table 5:** Important Aspects of the Randomized Clinical Trials Taken Into Account in the Analysis ADHD: attention deficit hyperactivity disorder; DSM: Diagnostic and Statistical Manual of Mental Disorders; GUAN-IR: guanfacine immediate-release; DMPH: dexmethylphenidate extended-release; COMB: combination; RCT: randomized controlled trial

First Author, Year	Study Type	Inclusion Criteria	Sample Size	Intervention	Outcomes
Sayer et al. (2016) [26]	RCT	7-14 years old participants with the primary diagnosis of ADHD using DSM-IV TR were included in the study.	207	The research spanned 1 year and 8 months and consisted of two distinct phases. Participants were randomly assigned to one of three study arms in a 1:1:1 ratio: 1) GUAN-IR - taken twice daily; 2) DMPH - taken once daily; 3) COMB - taken once daily. Additionally, participants were stratified into two age groups: 1) the younger group (7-10 years old) and 2) the older group (11-14 years old)	The study findings revealed that all three interventions were well-tolerated and considered safe. Furthermore, the cardiovascular changes observed in the GUAN-IR and DMPH study groups returned to their initial levels after consistent therapy, and no participant experienced any serious cardiovascular events.

Discussion

The treatment of ADHD often includes the use of stimulant medications, which has been supported by both clinical experience and scientific evidence [1]. Stimulants like methylphenidate (MPH) and amphetamines (AMP) have consistently shown effectiveness in reducing the core symptoms of ADHD including inattention, hyperactivity, and impulsivity [6]. These medications work by targeting specific neurotransmitters in the brain that are dysregulated in individuals with ADHD, specifically DA and norepinephrine (NE) [14]. By enhancing the availability of these neurotransmitters stimulants help improve attention, impulse control, and behavioral regulation [27]. However, it is important to note that there can be potential adverse effects associated with the use of stimulant medications for ADHD treatment [2]. While these medications are generally considered safe and well tolerated, it is crucial to be aware of the possible risks and complications they may pose [6]. In this discussion, we will analyze and synthesize findings from studies that examine the system-wide adverse effects of stimulant medications on children and adolescents diagnosed with ADHD. Although these effects are generally rare and mild in severity, it is still important to understand their potential implications.

Effect on the Cardiovascular System

All ADHD medications come with warnings on their package inserts about potentially serious adverse CV reactions and BP increases [28]. Stimulant medications work by increasing levels of neurotransmitters like NE and DA in the synaptic cleft. This increase in neurotransmitter concentration leads to heightened adrenergic and DA receptor stimulation [29,30]. Recent extensive research consistently indicates that both AMP and MPH have the potential to cause an increase in rRHR and systolic BP [31].

A meta-analysis conducted by Hennissen et al. found that medications like MPH, AMP, and atomoxetine resulted in statistically significant but slight increases in BP and HR. It’s important to note that the most noticeable alterations in BP and HR were observed over a short-term period suggesting a potential gradual return to normal levels over time [1]. This observation is supported by Omidi et al.’s 3-month follow-up study focusing exclusively on MPH. The study revealed a substantial increase in BP measurements without affecting the QT interval. Although these changes remained within the normal range during the study duration, the authors suggest there may be a potential for future hypertension [14]. Additionally, Torres-Acosta et al.’s study highlighted a connection between heightened HR and CVD, effectively demonstrating that MPH was linked to increased odds of prehypertension [22]. Therefore, it is crucial to closely monitor BP and HR, particularly in patients with pre-existing CV conditions [1]. However, in previous studies, there were no reports of significant CV events such as myocardial infarction, stroke, or sudden cardiac death [13,32-35].

A nationwide cohort study conducted in Denmark examined the CV safety of stimulants among children and adolescents with ADHD. In a comparison of ADHD prescription medication users versus nonusers (n = 8,300), there was a higher risk of adverse CVD events, such as arrhythmia (23%), cerebrovascular disease (9%), hypertension (8%), ischemic heart disease (2%), heart failure (2%), and pulmonary hypertension (1%) (adjusted hazard ratio: 2.3; 95% confidence interval [CI]: 1.2 to 4.8) in the users group [35]. It is important to understand that the relationship between stimulant treatment and CV adverse events is complex and depends on various factors such as time elapsed and dosage. However, several large cohort studies have not presented evidence of an increased risk of life-threatening CV events among children treated with stimulants compared to the general population [22]. Therefore, clear evidence regarding the causation of CV events by stimulant medications is lacking, and further research involving large cohorts is needed due to the low incidence of such events, making it difficult to demonstrate in randomized controlled trials [1].

A new approach has been proposed for managing ADHD in young people which involves employing different treatments together. However, we currently have insufficient research or data regarding the effectiveness of this approach. To investigate this further, Sayer et al. conducted a study examining the impact of combination therapies (specifically guanfacine immediate-release (GUAN-IR), dexmethylphenidate extended-release (DMPH), combination (COMB)) on young people with ADHD. The results revealed that there were no noteworthy CV changes observed during both the short-term and long-term phases of the combined treatment. The study also suggested that any minor CV changes observed during short-term treatment tended to return to normal over time [26]. Notably, similar to the previous studies, these medications had varying effects; for instance, GUAN-IR led to minor decreases in HR and BP [36-38]. Moreover, combination treatment involving guanfacine extended-release (GUAN-XR) and stimulants exhibited the potential to mitigate the HR elevations caused by stimulant medications [39]. However, it is crucial to conduct further research using larger sample sizes and longer durations of treatment to strengthen these conclusions and explore the day-to-day variability of CV parameters.

Effect on the Central Nervous System

Stimulants have various ways of affecting the central nervous system (CNS) by interacting with neurotransmitters and receptors in the brain. This leads to increased neurotransmission and activation of pathways related to attention, focus, and arousal [19]. When used under proper medical supervision, long-term treatment with MPH is both safe and efficient in managing ADHD symptoms in children [40]. In a systematic review conducted by Krinzinger et al., it was found that there is variability in the methodologies and study designs used to investigate the long-term effects of MPH treatment [16]. However, the majority of studies indicate that extended use of MPH does not cause anxiety and irritability, especially for individuals beyond preschool age. Furthermore, evidence from studies examining substance abuse suggests that long-term MPH use has no negative outcomes. However, caution should be exercised when prescribing MPH to high-risk adolescents to minimize potential abuse [16]. Regarding depressive symptoms, the collective findings of the studies indicate that extended treatment with MPH generally leads to positive outcomes, including a decrease in suicidal tendencies among individuals with ADHD [16].

Although there is evidence of an increased risk of psychosis and tics, reported cases have shown that these symptoms typically resolve upon discontinuation of the medication [25]. Overall, multiple studies support the safety of long-term MPH use regarding psychosis and tics. However, caution is advised when prescribing MPH to individuals who are susceptible to psychosis or tics, as indicated by case reports [16]. In a cohort study carried out by Moran et al., which included 221,846 adolescents and young adults diagnosed with ADHD and prescribed either MPH or AMP, the researchers discovered that among these individuals, 343 patients were diagnosed with psychosis. As a result, these patients were subsequently prescribed antipsychotic medication. The percentage of patients experiencing a psychotic episode was 0.10% in the MPH group and 0.21% in the AMP group. The incidence of a psychotic episode was also higher in the AMP group compared to the MPH group, with rates of 2.83 per 1000 person-years and 1.78 per 1000 person-years, respectively [19].

To summarize, based on this information, it is estimated that around one in every 660 patients receiving stimulant prescriptions for ADHD may develop new-onset psychosis. However, the risk is approximately twice as high in patients initiating AMP treatment compared to those starting MPH treatment [19]. While there isn’t enough evidence to definitively determine whether MPH is linked to the emergence of psychotic symptoms during treatment, available information suggests that psychotic symptoms may occur in approximately 1.1-2.5% of individuals undergoing MPH treatment. Therefore, clinicians should remain vigilant and acknowledge the potential occurrence of psychotic symptoms during MPH treatment [25].

Effect on the Gastrointestinal System

The gastrointestinal side effects of stimulant medication in ADHD patients can be attributed to various factors. First, these medications can increase stomach acid production leading to symptoms like heartburn and indigestion. Second, they often reduce appetite, causing stomach pain, nausea, and vomiting. Third, stimulant medications can affect the normal movement of the gastrointestinal tract resulting in delayed gastric emptying, bloating, and constipation. Additionally, the gastrointestinal lining may become irritated leading to inflammation and abdominal discomfort [41-43]. A systematic review conducted by Holmskov et al., which included randomized parallel and cross-over trials, comparing MPH to placebo or no intervention, indicated a potential for decreased appetite, weight loss, and increased abdominal pain. However, the study did not find any significant difference in the likelihood of gastrointestinal adverse events based on the method, dosage, or duration of MPH usage. Moreover, both immediate-release and extended-release forms of MPH had similar risk profiles [23]. Further high-quality randomized clinical trials are needed to better understand the risks and optimize treatment strategies, including longer durations, well-documented dosage and type of MPH, and systematic measurement of gastrointestinal adverse events.

Effect on the Ocular System

Stimulant medications used in the treatment of ADHD can cause various ocular side effects in patients. Some examples of these side effects are blurred vision, dry eyes, pupil dilation, changes in intraocular pressure, and eye redness [20]. The exact mechanisms behind these ocular effects are not fully understood, but it is believed that the medications’ impact on neurotransmitter levels, particularly DA and NE, may play a role [44]. These alterations in neurotransmitter activity can affect how ocular structures and pathways function ultimately leading to visual disturbances. Bingöl-Kiziltunc et al. aimed to assess how methylphenidate hydrochloride affected functional and structural ocular parameters in ADHD patients. Their findings indicated that the majority of patients exhibited blue-purple color weakness initially, which could be attributed to the impact of DA on color vision, particularly affecting S cones. After one year of MPH treatment, they observed structural changes such as an increase in myopic values of static retinoscopy and axial length and a decrease in hyperopic values of cycloplegic retinoscopy. The only noticeable change in functional parameters was a decrease in the ability to accommodate [20].

In a case report by Soyer et al., they documented the case of a nine-year-old child who experienced a significant decline in visual acuity as a result of an accommodation disorder after being treated with MPH and lisdexamfetamine [45]. Similarly, Bingöl-Kiziltunc et al. found a significant difference in accommodation capacities before and after 12 months of MPH use. While these children did experience a reduction in their ability to accommodate due to the medication, they did not report blurry vision because they were still able to adjust their focus. These findings suggest that children who undergo long-term MPH treatment may eventually experience blurred vision because of declining accommodation capacities [20]. Additionally, it is important to consider the potential presence of glaucoma when using stimulant medications. These medications have a mild anticholinergic effect and hinder the reuptake of noradrenaline, which can contribute to the development or worsening of angle closure glaucoma [46]. Furthermore, they have also been known to temporarily increase intraocular pressure without causing angle closure [47]. In another case report by Lu et al., it was reported that a 10-year-old boy developed bilateral open-angle glaucoma while being treated with MPH supporting the literature [48].

General Side Effects

The side effects of stimulant medications in individuals with ADHD are primarily caused by the impact these medications have on the central nervous system and other physiological processes [16]. Common side effects include decreased appetite, trouble sleeping, headache, and stomach ache. Some people may also experience mood swings, irritability, or anxiety [6].

A systematic review conducted by Storebø et al. examined non-randomized studies on the adverse events of MPH in children and adolescents. The review included 260 relevant studies with a total of 2,283,509 participants worldwide. It found that serious adverse events were 1.36 times more likely to occur in participants using MPH compared to controls in the comparative cohort and patient control studies. However, the review acknowledged limitations such as inconsistent reporting and limited data on comorbidities and medication use [24]. In a systematic review led by Kamal et al., the influence of MPH on ADHD-affected children and adolescents in Qatar’s public schools was examined [6]. The results highlighted that the administration of MPH notably enhanced these individuals’ academic achievements, behavioral attributes, and familial harmony, mirroring the conclusions of corresponding global research [49]. Nonetheless, it’s significant to emphasize that a substantial 72% of the participants acknowledged experiencing side effects post-MPH consumption, irrespective of their use of extended-release or immediate-release formulations. Intriguingly, female participants manifested a higher frequency of side effects relative to their male counterparts. Moreover, individuals initiated on a blended regimen of both drug variants presented a heightened prevalence of side effects compared to those on a singular medication type [6].

Growth and Bone Age

One area of concern regarding stimulant medication like MPH is its potential impact on growth and bone age in individuals with ADHD. Research suggests that the use of stimulants may result in a temporary decrease in growth velocity during the initial year of treatment [50]. However, most children eventually catch up to their expected growth trajectories. The long-term impact on final adult height remains uncertain, with conflicting findings reported [51]. When it comes to bone age, stimulant use may cause minor delays in skeletal maturation, however, these delays are reversible and generally not clinically significant [21]. In a study conducted by Carucci et al., the long-term effects of MPH use on height and weight were investigated. It was found that children and adolescents may experience slight impacts on their height and weight due to long-term MPH treatment for ADHD. Over two years, the group-level effects indicated a potential reduction in height gain of around 1.39 cm and weight gain of about 1.96 kg. Nonetheless, these symptoms are usually temporary and return to normal over time [2]. Factors such as decreased appetite, endocrinological factors, and medication dosage were discussed as possible reasons for the medications’ effects on growth [21,50,52-55].

On the other hand, a study conducted by Poulton et al. examined the immediate and long-term effects of medical treatment for ADHD on growth and bone age [21]. It was found that treated children showed slower growth than untreated children, but this slower growth did not correspond to a delay in bone age, consistent with findings from three cross-sectional cohort studies [56-58]. Treated children were maturing more quickly than expected for their growth in height. The study attributed the attenuated height velocity to changes in energy balance caused by appetite suppression and identified baseline energy stores as a predictor for changes in height [21]. Overall, both studies highlight the need for further research to better understand the long-term effects of stimulant medication on growth in individuals with ADHD, but they approach the topic from different perspectives.

Limitations

The systematic review has certain limitations that must be taken into account when interpreting the results. The use of specific eligibility criteria, such as excluding studies published in languages other than English or before 2013 and studies that have restricted access may have introduced selection bias. Additionally, focusing only on studies including individuals aged 0 to 25 years and including both review articles and research articles could introduce bias in the findings. There is also a possibility of missing relevant studies despite employing various search strategies across multiple databases and using different keywords. Moreover, relying solely on published studies may lead to publication bias. Therefore, it is crucial to interpret the findings and draw conclusions from this systematic review while considering these limitations. Although this review adheres to PRISMA 2020 guidelines and provides a comprehensive analysis of adverse effects in the ADHD population, we need to be cautious about its limitations.

## Conclusions

In conclusion, this systematic review offers an extensive analysis of system-wide adverse effects associated with stimulant medications in children and adolescents with ADHD. While these effects are generally rare and mild, it is important to be aware of their potential implications. When examining CV effects, stimulant medications were found to slightly increase BP and HR but did not result in serious CV events.

The impact on the central nervous system was also assessed, indicating that long-term MPH treatment appears to be generally safe but carries a higher risk for psychosis when using AMP. However, there is limited evidence regarding the neuropsychiatric effects and cognitive functioning of long-term MPH treatment. Hence, further research is needed in these areas. We did not find significant adverse effects of MPH on academic performance, behavior, or family well-being in the existing literature. The side effects are mostly mild and can be managed easily. However, caution is necessary and it is recommended to monitor the situation carefully. In conclusion, this systematic review highlights the significance of taking into account the potential adverse effects of stimulant medications when evaluating the benefits of treating ADHD. It also urges further research to enhance our comprehension of these effects and their long-term consequences.
